# Optimization of the scan length of head traumas on the pediatric and adult CT scan and proposition of a new acquisition limit

**DOI:** 10.1038/s41598-021-90529-4

**Published:** 2021-05-26

**Authors:** Fotso Kamdem Eddy, Samba Odette Ngano, Fotue Alain Jervé, Abogo Serge

**Affiliations:** 1 Unité de Recherche de la Matière Condensée, d’Electronique et de Traitement du Signal, Department of Physics, Faculty of Science, University of Dschang, Dschang, Cameroon; 2 Department of Radiography, Yaoundé General Hospital, Yaoundé, Cameroon; 3 Department of Radiology, National Social Insurance Fund Hospital, Yaoundé, Cameroon

**Keywords:** Biophysics, Cancer, Physics

## Abstract

To propose a new method of reducing the scan length of head trauma while keeping the diagnostic efficiency of the examination in order to develop DRL in an African context. This is a retrospective single-center study including 145 patients who had cranial examinations on a 64-barettes scanner. All head trauma cases were selected. The interpretations of these CT scanners by the three radiologists of the service were noted to determine the acquisition limit. All patient acquisition lengths have been recorded. The acquisition limit for head trauma ended in clinical routine at cervical spine 4 (C4). The average scan length was 23.03 cm. Out of the CT scan results for 145 patients, only 2 (1.37%) had a C3 level cervical spine fracture and 2 (1.37%) at C4. By respecting the principles of radiation protection, this result has shown us that it is possible to limit the acquisition length of the CT scanners indicated for head trauma. The limit of the optimized scan length that we proposed is at cervical spine 2 (98.62%). Now, all head trauma are limited on cervical vertebra 2 in our hospital. The use of this new method is beneficial when the clinical indication of the examination and the type of trauma (multi-trauma) are taken into account. Based on the principles of radiation protection and the clinical indication for the examination, reducing the scan length from C4 to C2 is an effective way to reduce the dose absorbed by the patient.

## Introduction

Among medical imaging techniques, Computed Tomography (CT) is the first-line imaging modality for the diagnosis of post traumatic cranio-encephalic lesions. Physical and financial inaccessibility are the main limitations of Magnetic Resonance Imaging (MRI) in most of these hospitals to avoid ionizing radiations. The price to pay for this frequent use is the increase in doses delivered to patients. The substitution of the CT scan by Magnetic Resonance Imaging (MRI) or ultrasound is a method of reducing the doses absorbed to patients and is already mandatory in pediatrics^1,2^. The long life expectancy of pediatric patients is likely to increase their irradiating doses received^3,4^.

Actually, there is currently no pediatric and adult regulatory Diagnostic Reference Level (DRL) in Cameroon to standardize protocols in hospitals. Published studies have shown that it is possible to reduce the dose using the Iterative Reconstruction (IR) algorithm^5–11^, by lowering the tube current^12–14^, increasing the pitch^15^, reducing the tube tension^5,6^ and using modulation automatic tube current^16^. Other studies have also shown that radiation doses can be reduced by studying the acquisition length^17–20^. Reducing the scan length is an effective means to reduce the CT scan dose. The limitation of the acquisition lengths can be correctly realized only in knowledge of the clinical indications of the request for examination. There are currently no regulatory scan lengths in this country and in black Africa. Identifying the limit of acquisition for head trauma is more complicated.

In this hospital, we reduce the acquisition coverage by trying to locate the cervical spine 4 on the scoot view image for all head trauma. However, the position of the trauma is often difficult to identify and to our knowledge, the effectiveness of this method has not yet been evaluated. Find a more reliable method to reduce the acquisition length in order to avoid the exposure of the thyroid to ionizing radiation, allowed us to evaluate the method used in this hospital to propose an acquisition limit. The legislative and regulatory frameworks in terms of scan length on patients are either non-existent or implemented in an approximate manner in our country.

As part of a project to improve the practices of pediatric CT scan, this work aimed at proposing a new method to reduce the scan length of head trauma while keeping the diagnostic efficiency of the examination.

## Material and method

### Patient characteristics

Local ethics committee of the National Social Insurance Fund Hospital authorities approved this study. For the retrospective nature of this study, informed consents for participating in this study was waived by the Institutional Review Board of the National Social Insurance Fund Hospital. Method was carried out in accordance with relevant guidelines and regulations. All patients who met the clinical criteria for head trauma were selected. Between September and December 2019, we identified 160 patients. The age group used in this retrospective mono-centric study consisted of two sexes between the ages of 5 and 75. For all patients, the following variables were collected: sex, age (years), weight (kg), height (m), Volume CT Dose Index (CTDIvol), Dose Length Product (DLP), indication of the examination, the interpretation of the examinations by the three radiologists of the service, the tube current–time product (mAs) and the scan length.

### Scan protocol

All the examinations were carried out on the same Neusoft 64-Barettes multi-cut CT scan manufactured in September 2017 and put into operation in April 2018. The examination protocols were standardized. The acquisition length was changeable and adapted to the patient’s size. All CT scanners were produced with a range of 100 to 120 kV. Automatic load modulation was adjusted to attenuate the anatomical area to be explored with a tube current–time product ranging from 149 to 400 mA. The post-acquisition CT scanner presented a dosimetric report on which appeared the administered doses expressed in CTDIvol and DLP for each acquisition and for the entire examination. The results of the examinations were noted after interpretation by the three radiologists.

### Scan length evaluation

The scan lengths expressed in mm corresponded to the difference between the first and the last section of the acquisition (beginning of the skull to the cervical spine 4 (C4)).

### Statistical analysis

For all examinations and for a single acquisition, the interpretations of the examinations and the scan length were recorded on the console and processed on Microsoft Excel. We calculated the average values of the scan lengths of all head traumas selected. All data was analyzed on Microsoft Excel 2016.

## Results

The high tube voltage ranged from 110 to 120 kV. 100% of acquisitions were in helical mode. The tube current–time product varied between 25 and 260 mA. The iterative reconstruction technique is used. The slice thickness range is 0.625 and the collimation is 0.625 mm. 145 patients participated in this study (80 children for age < 18, 65 adults for age < 70). We had 50 girls and 95 boys in total. In addition, we study blunt or non-penetrating trauma. The clinical neurological findings that the clinicians used to request the CT head-scan were loss of consciousness and cephalalgia post RTA (Road Traffic Accident). We also had 30 EDH (Epidural Hematoma), 47 SDH (Subdural Hematom), 2 SAH (Subarachnoid Hemorrhage) and 66 fractures. We didn’t find the participants with spinal cord injury.

**Figure 1:**
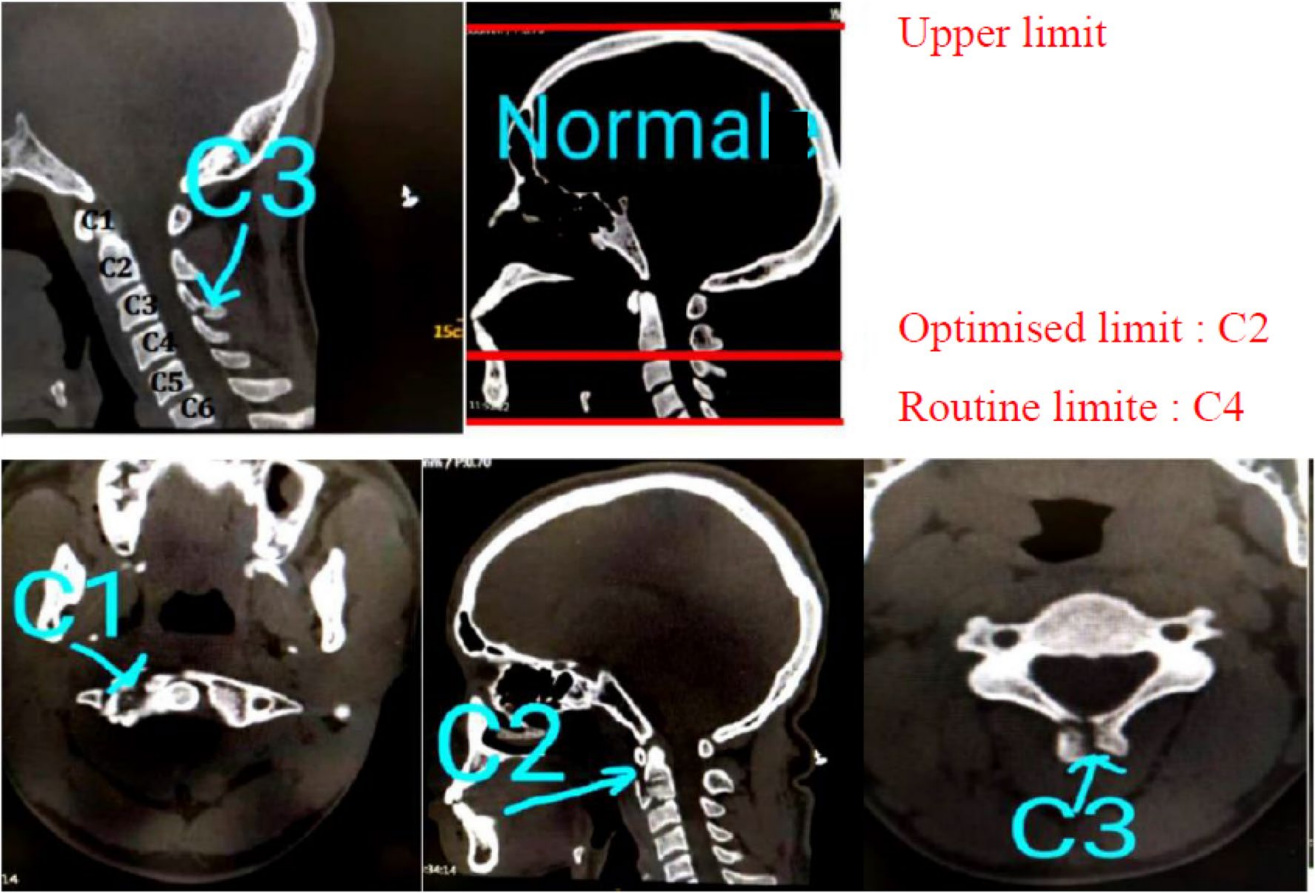
Optimized acquisition limit and position of fractures.

All head trauma scan lengths are routinely delimited to the cervical spine 4. This shows that all head traumas are cranial-cervical spine examinations. The average scan length of all head traumas is 23.03 Cm (modifiable). The results of the interpretations of the examinations by the three radiologists in the department showed that on the 145 patients selected, only 4 had a cervical spine link in C3 and C4. Which gives us respectively a percentage of 1.37%. This proves that for cases of head traumas, it is possible to limit acquisition coverage to the level of the cervical spine 2 (98.62%) (Fig. 1). By respecting the principles of radiation protection, we propose to limit acquisition coverage to C2 to protect the thyroid against these Ionized Radiations (IR) (and diffused radiation).

Table 1 describes the characteristics of acquisition coverage for all selected traumas. The acquisition length should be centered on the area of interest. In our study, we proposed a new method to place the acquisition limit for head trauma.

**Table 1:** Characteristics of acquisition coverage for all selected trauma.

Scan length	Optimized limit (C-spine C2)	Intermediate limit (C-spine C3)	Routine limit (C-spine C4)	Average length
Population (%)	143 (98.62)	2 (1.37)	2 (1.37)	23.03 cm

This acquisition limit is made up of the beginning of the skull and the end of the cervical spine 4. With our acquisition limit, we were able to reduce the number of cervical vertebra in the acquisition length by 50%. Even if our method has shown that it is possible to have injuries in the cervical vertebra C3 (1.37%) and C4 (1.37%) (Fig. 1), their percentage remains low (Table 1), this seems acceptable in current clinical practice. We therefore believe that the new method that we are proposing appears to be a good compromise by allowing a reduction in the acquisition length greater than the method applied in routine. Now, all head trauma are limited on cervical vertebra 2 in our hospital.

For adults, best practice is to separately scan the head to C1 then wait 15 s and scan the C-spine from C1 to T1 (thorax 1) to avoid missing any injury. For children the principle is to focus on the head CT scan as the incidence of Cervical spine injury is very low and a screening lateral plain film is acceptable.

## Discussion

This work presents the current practice of CT scan of the head trauma of patients in one hospital in the west Cameroun. It is most active in terms of pediatric and adult CT scan in the West Cameroon. The main finding of this study was to propose the limit of the optimized scan length at cervical spine 2 (98.62%). In routine, the protocol we use for all head trauma involves scanning from the head to the cervical spine 4. So here, we demonstrate this protocol can be reduced to cervical spine 2 for classical head trauma. It is possible that the injury is beyond this coverage (1.37% for this study) or it is possible that the injury is in the cervical spine 5, 6 or 7 but this depends on the severity of the accident and these are rare cases. We also limited ourselves to the indication of the prescribing physician for head trauma. It is important that clinicians ordering CT should do a careful clinical examination of the cervical spine and inform the radiologists of the findings. This will limit the scan length or the number of examinations in the patient. The results observed prove that the scan lengths are not optimized. The absence of regulatory scan lengths in the medical imaging services of this country leads to the overexposure of certain radiosensitive organs to IR. For radiation protection and patient protection against IR, limiting patient exposure doses is a priority. Scan length plays a big role in optimizing radiation doses. It is directly related to the radiation dose delivered by the CT scan and defines the region of patient exposure. Optimizing scan length is a simple, easy and beneficial technique for reducing radiation doses to all patients. Most radiosensitive organs are included in and exposed to the acquisition coverage (including the dose of scattered radiation).

The scan length must be strictly adapted to the clinical indication of each patient and must be limited to the area of interest, previously indicated by the prescribing physician. Optimizing the acquisition length therefore reduces the radiation dose to the CT scan. There is however the problem of diagnosis and principle of radiation protection in this concept of scan length.

Ethical rules suggest that any injury with a risk of more than 1% should be identified. It is true that “if you scan the head for BLUNT trauma you should scan the whole spine” but we have studied cases of mild trauma. Multiple trauma cases were excluded. In general for these polytrauma victims, the clinicians ask to scan the whole body. This is normal but for mild trauma we suggest it is not necessary to do so. This is why the results of our examinations carried out from C2 to C4 were satisfactory. We believe that, for a head trauma whose result of the examination presents Epidural hematoma, Subdural hematoma and skull fractures, it is not necessary to scan the whole spine but on the other hand if the spinal cord is affected, it is imperative to go up to the whole spine. This is not the case in this research. We did not have a spinal cord injury.

All medical imaging technicians delimited as they learned at the training school. Practices are improving every day through published scientific discoveries, but most of these medical imaging technicians are not informed of these discoveries. They apply the same protocol to limit the scan lengths they learned during their academic internship. Clinicians must first properly diagnose patients take into account the circumstances of the head trauma to indicate the right area of interest. Displaying optimized scan lengths in the control room will help to control protocol optimization and compare clinical practices. These lengths will reduce the exposure of parts of patients who are unnecessary in examinations. Medical imaging technicians rely on the notion of diagnosis to increase the scan length (the pathology may be beyond the area of interest). Which brings us back to the notion of uncertainty. This method obviously avoids having to do another exam but also helps to expose other parts of the body. As far as we are concerned, the choice of patient protection against IR is a priority. It therefore comes down to asking us questions about the concepts of clinical routine used in our hospitals.

In Cameroon, due to the absence of a legislative framework in this area, national Diagnose Reference Level (DRL) are not established and made available to the public. In addition, Cameroon does not have a quality control structure to verify the compliance of the radiation doses delivered to patients. Despite the presence of a law regulating radiation protection and a national radiation protection agency in Cameroon^21^, the limitation of acquisition coverage remains uncertain in each hospital. The American Association of Physicists in Medicine (AAPM) Reports 111 and 204 have shown that the radiation dose from patient CT scanners depends on their size and length of scan^18^. Published studies have shown that reducing acquisition coverage^22,23^ is a simple and effective way to limit the dose of patients and is recommended in clinical practice. In addition, by optimizing coverage, it is also possible to reduce the exposure of radiosensitive organs^24^. Corwin et al. found that a 24% reduction in scan coverage corresponded to a 23% dose reduction^22^. A shorter scan length means a lower dose if all the other acquisition parameters are kept constant: “The smaller the exposed area, the smaller the dose”^25^.

Furthermore, it would be imperative to introduce in Cameroon, comprehensive legislation for the radiation protection of patients in order to have national pediatric and adult DRL. Create regional quality control structures and train medical physicists in radiology for public^26^ and private hospitals to improve practice in our country. In addition, it would be necessary to set up compulsory continuing education for the medical and paramedical staff concerned^27^.

## Conclusion

To our knowledge, this study is the first to focus on the evaluation of patient acquisition lengths in diagnostic CT practice in Cameroon. This is a retrospective and evaluative study of head trauma in a single hospital. It will serve as a guide for other hospitals in the country. In clinical practice, medical imaging technicians will now rely on the clinical indication of the trauma and the causes of the accident before limiting the length of patient acquisition. According to the principles of radiation protection, reducing the scan length from C4 to C2 is an effective way to reduce the dose absorbed by the patient.
